# Chlamydia diagnosis rate in England in 2012: an ecological study of local authorities

**DOI:** 10.1136/sextrans-2015-052441

**Published:** 2016-08-31

**Authors:** Lakshmi Chandrasekaran, Bethan Davies, Jeffrey W Eaton, Helen Ward

**Affiliations:** 1 School of Medicine, Imperial College London, London, UK; 2 Department of Infectious Disease Epidemiology, School of Public Health, Imperial College London, London, UK

**Keywords:** CHLAMYDIA TRACHOMATIS, SCREENING, PUBLIC HEALTH, SEXUAL HEALTH, PROGRAM EVALUATION

## Abstract

**Objectives:**

Local authorities (LAs) in England commission chlamydia screening as part of the National Chlamydia Screening Programme. It is recommended that LAs achieve a chlamydia diagnosis rate of ≥2300 cases per 100 000 population aged 15–24. We describe national patterns in attainment of the chlamydia diagnosis rate recommendation and possible implications of using it to measure LA-level performance.

**Methods:**

We used publicly available data sets from England (2012) to explore the association between LAs attaining the recommended chlamydia diagnosis rate and population size, socioeconomic deprivation, test setting and sex.

**Results:**

We used data from 1 197 121 recorded chlamydia tests in females and 564 117 in males. The chlamydia diagnosis rate recommendation was achieved by 22% (72/324) of LAs overall (43% female population; 8% male population). LAs in the highest deprivation quintile were more likely to reach the recommendation than those in the least-deprived quintile for both sexes (women: unadjusted prevalence ratio (UPR) 7.43, 95% CI 3.65 to 15.11; men: UPR 7.00, 95% CI 1.66 to 29.58). The proportion of tests performed in genitourinary medicine clinics was negatively associated with attainment of the recommended diagnosis rate (UPR 0.95, 0.93 to 0.97).

**Conclusions:**

Chlamydia diagnosis rate recommendations that reflect local area deprivation (as a proxy for disease burden) may be more appropriate than a single national target if the aim is to reduce health inequalities nationally. We suggest LAs monitor their chlamydia diagnosis rate, test coverage and test positivity across a range of measures (including setting and sex) and pre/post changes to commissioned services. Critical evaluation of performance against the recommendation should be reflected in local commissioning decisions.

## Introduction

Chlamydia infection, caused by *Chlamydia trachomatis*, is the most commonly diagnosed STI in England.^1^ National surveys performed in the UK have estimated that the prevalence of infection is highest among young women of 18–19 years (4.7%; 95% CI 2.5% to 8.6% in 2010–2012) and men of 20–24 years (3.4%; 2.2% to 5.2%).^2^ A National Chlamydia Screening Programme (NCSP) was introduced in 2003 in England by the Department of Health. The aim was to control chlamydia prevalence, transmission and reduce reproductive tract morbidity. The NCSP recommends opportunistic screening for sexually active males and females under 25 years, annually or after a partner change, whichever is more frequent. Testing occurs in a variety of settings, including genitourinary medicine (GUM) clinics, general practice, schools and youth centres.

It is difficult and costly to obtain prevalence estimates for chlamydia. Therefore, NCSP performance monitoring has focused on process measures. The previous annual target of 35% coverage of 15–24 year olds was criticised because it did not encourage services to target individuals most at-risk of infection.^3^ A novel indicator, the chlamydia diagnosis rate, was introduced in 2013 by Public Health England (PHE) within the Public Health Outcomes Framework. PHE recommends that each local authority (LA) (governmental administrative areas of England) achieve a chlamydia diagnosis rate of ≥2300 cases per 100 000 residents aged 15–24 years per year.^4^

The chlamydia diagnosis rate is calculated by dividing the number of unique positive tests performed within the NCSP by the total population aged 15–24 years. This is equivalent to multiplying test coverage (the proportion of the population screened) with positivity (the proportion of tests that are positive) and, therefore, it encourages targeting resource towards at-risk individuals and community-wide screening.^4^ ^5^ There are currently no published studies that explore the relationship between chlamydia diagnosis rate and external factors. But coverage and positivity are associated with a multitude of factors that differ across LAs, including gender,^2^ population size or urbanisation,^6^ socioeconomic status^7^ ^8^ and type of testing facility. We describe patterns in the national attainment of the chlamydia diagnosis rate indicator and the possible implications of using it to measure LA-level performance.

## Methods

We used the 2012 NCSP data set (NCSP website 05/03/2014), which contains all chlamydia tests performed and all diagnoses reported in people aged 15–24 in England during 2012 by LA.^9^ The data set also contains the location where tests were performed (GUM clinics or non-GUM settings) for the combined population (male and female) and the 15- to 24-year-old population size by sex (from 2011 census). We used the Indices of Multiple Deprivation 2010 data set (which contains a deprivation score for each Lower Super Output Area, a subdivision of LAs that have a population of 1000–3000) to calculate a weighted-average socioeconomic deprivation score for each LA.^10^

Separately for the male and female population in each LA, we calculated the chlamydia diagnosis rate per 100 000 residents aged 15–24. This calculation assumes that each test is from a unique person. We categorised LAs into those that achieved and those that did not achieve the recommended chlamydia diagnosis rate. We calculated unadjusted prevalence ratios (PRs) to explore the relationship between attaining the recommendation and population size, and deprivation by sex and then the proportion of tests in GUM clinics for the overall population. We carried out a χ^2^ test for trend in R (V.3.2.4) to explore the evidence for a trend in the proportions attaining the recommendation according to categories of IMD and population size. Ethics committee approval was not sought for this study.

## Results

The 2012 NCSP data set included 1 197 121 recorded chlamydia tests in females and 564 117 in males aged 15–24 years in 326 LAs. We excluded two LAs (NCSP data set recorded ‘<5’ for key variables). For the overall population, the chlamydia diagnosis rate recommendation was achieved by 72 of 324 (22%) LAs, and the mean chlamydia diagnosis rate for England was 1800 per 100 000. For women, nearly half (139/324, 43%) of all LAs achieved the recommendation with a mean of 2338 per 100 000 (table 1). For men, <10% (26/324, 8%) of LAs attained the recommendation with a mean diagnosis rate of 1244 per 100 000.

**Table 1:** Coverage, positivity, chlamydia diagnosis rate and unadjusted PRs for the association between achieving a chlamydia diagnosis rate of ≥2300 cases per 100 000 by quintile of socioeconomic deprivation (IMD) and sex for LAs in England in 2012

IMD quintile	No of LAs	LAs meeting recommendation (%)		Coverage (%)		Positivity (%)		Chlamydia diagnosis rate per 100 000		Unadjusted PR (95% CI)
		N	Per cent	Mean	SD	Mean	SD	Mean	SD	
Women										
1*	65	52	80.00	39.65	11.91	7.97	1.09	3176	1128	7.43 (3.65 to 15.11)
2	65	36	55.38	37.36	14.43	7.27	1.40	2651	946	5.14 (2.47 to 10.70)
3	64	27	42.19	32.84	16.19	7.31	1.36	2357	1147	3.92 (1.84 to 8.35)
4	65	17	26.15	26.54	7.63	6.76	1.18	1791	596	2.43 (1.08 to 5.46)
5†	65	7	10.77	27.34	16.62	6.43	1.16	1713	966	Reference
Men										
1*	65	14	21.54	19.22	8.47	9.90	2.98	1772	756	7.00 (1.66 to 29.58)
2	65	7	10.77	16.55	9.45	9.52	2.69	1434	586	3.50 (0.76 to 16.22)
3	64	5	7.81	14.18	10.11	9.19	3.15	1175	645	2.54 (0.51 to 12.61)
4	65	0	0	11.11	4.89	8.85	2.31	921	291	‡
5†	65	2	3.08	12.47	8.82	8.36	2.36	919	448	Reference

*IMD 1 is the most-deprived quintile.

†IMD 5 is the least-deprived quintile.

‡Data omitted because no LAs in IMD quintile 4 met chlamydia diagnosis rate recommendation.

IMD, index of multiple deprivation; LA, local authority; PR, prevalence ratio.

LAs in the most socioeconomically deprived quintile were more likely to achieve the recommendation than those in the least-deprived quintile for both women (PR 7.43, 95% CI 3.65 to 15.11, χ2 test for trend p ≤ 0.001) and men (PR 7.00, 1.66 to 29.58, χ2 p ≤ 0.001). In the overall population, the proportion of tests carried out in a GUM setting was negatively associated with attaining the recommendation (PR 0.95 (0.93 to 0.97)). LAs with larger female populations were more likely to attain the recommended chlamydia diagnosis rate than those with a smaller population (χ^2^ test for trend p≤0.0001), but there was no clear association between achieving the recommendation and population size for men (p=0.2619, see online supplementary table S1).

10.1136/sextrans-2015-052441.supp1 Supplementary table

## Discussion

The chlamydia diagnosis rate recommendation of ≥2300 cases per 100 000 population aged 15–24 was more likely to be attained by LAs with higher levels of socioeconomic deprivation and those where a higher proportion of tests were performed outside the GUM setting. It was also more likely to be reached in the female population compared with the male population. While LAs can influence the location of chlamydia testing through commissioning arrangements, the association between deprivation and attaining the recommended level suggests that using the chlamydia diagnosis rate to compare performance across LAs is difficult.

To our knowledge, this is the first study that explores the novel chlamydia diagnosis rate recommendation for the NCSP in England. We have performed a secondary analysis of publicly available data sets and our findings are limited by the quality of these data. However, the Chlamydia Testing Activity Dataset used in this analysis has considerably improved the quantity and utility of NCSP data.^11^ It includes all chlamydia tests performed in National Health Service or LA-commissioned laboratories; residence details to allocate cases to LAs; and it can be linked to GUM data to reduce ‘double-counting’ cases diagnosed in non-GUM settings but referred to GUM.

It was not possible to adjust the population denominator by the proportion who are sexually active and if this parameter varies by LA it could bias our findings. In addition, the available data assume that each test is a unique person. It is likely, given the policy recommendation for repeat testing following a positive test or change of sexual partner, that a proportion of people are tested more than once per year and, therefore, estimated coverage is likely to overestimate true population coverage. The weighted-average deprivation scores we used compare area rather than individual-level deprivation; therefore, LA values may not reflect the individuals participating with chlamydia testing, and individual-level data are needed to fully understand the local picture.

The introduction of the chlamydia diagnosis rate indicator is a shift from a process measure of 35% coverage to a consideration of whether testing is achieving the aim of identifying asymptomatic infections. Our interpretation of this study assumes that achieving this recommendation is a marker for ‘better’ chlamydia control activity. But the impact of diagnosing asymptomatic infections in men and the relationship between testing rates and population prevalence of infection are not known. Further research is needed to determine how the chlamydia diagnosis rate is expected to change to reflect adequate control of the infection.

Our findings suggest that using the chlamydia diagnosis rate as a national public health indicator may be compromised by the influence of external factors. More affluent LAs were less likely to attain the recommendation compared with the most-deprived areas. This study was not able to explore the mechanism of this association but potential causes include more appropriate targeting of testing or a higher disease burden in deprived areas. There is a well-documented association in the UK between increasing area-level deprivation and chlamydia infection (combined OR from meta-analysis of three studies 1.76, 95% CI 1.15 to 2.71).^8^

The negative association between the proportion of tests in GUM clinics and reaching the chlamydia diagnosis rate recommendation may suggest the need for more testing outside of this setting, particularly in the least-deprived areas. A potential explanation for this is that chlamydia (unlike gonorrhoea and other STIs) is not typically concentrated in higher risk individuals who may preferentially present to GUM clinics rather than primary care. Further analysis of chlamydia diagnosis rate in GUM clinics, compared with non-GUM settings, by sex, could help commissioners to determine the ideal balance of testing locations.

We observed a marked difference in the mean chlamydia diagnosis rate by sex. This may reflect the higher prevalence of chlamydial infection in young women shown by the recent UK National Survey of Sexual Attitudes and Lifestyles (prevalence in women 16–24 years was 3.1% (2.2–4.3); 2.3% (1.5–3.4) in men^2^) or it may highlight a need to encourage screening among men.

### Implications for policy makers

Recommending a consistent chlamydia diagnosis rate to LAs with potentially differing local burdens of infection^8^ may result in an overinvestment of resource relative to health need in affluent areas. Therefore, adjusting the recommendation by area socioeconomic status, as a proxy for local disease burden, may be more appropriate than a single national recommendation.

We suggest that LAs are encouraged to evaluate their chlamydia diagnosis rate attainment, test coverage and positivity across a range of measures (including service, sex, geographic area (lower super output area)) and before and after changes to commissioned services. Critical evaluation of LA performance against the recommendation should be reflected locally in commissioning decisions and nationally in subsequent versions of the Public Health Outcomes Framework.

## References

[1] Public Health England. Sexually transmitted infections and chlamydia screening in England, 2014. Health Protection Report: Weekly Report. 23/06/2015 ed: PHE, 2013.

[2] Sonnenberg P, Clifton S, Beddows S, et al Prevalence, risk factors, and uptake of interventions for sexually transmitted infections in Britain: findings from The National Surveys of Sexual Attitudes and Lifestyles (Natsal). Lancet 2013;382:1795–806. 10.1016/S0140-6736(13)61947-9 24286785 PMC3899025

[3] National Audit Office. Department of Health Young people's sexual health: the National Chlamydia Screening Programme. National Audit Office, 2009.

[4] Public Health England. Public Health Outcomes Framework (2013–16). 10 December 2015. https://www.gov.uk/government/publications/healthy-lives-healthy-people-improving-outcomes-and-supporting-transparency.

[5] National Chlamydia Screening Programme. Public Health Outcomes Framework: Annual Chlamydia Diagnosis Rate (15–24-year-olds) Frequently Asked Questions (FAQ). Secondary Public Health Outcomes Framework: Annual Chlamydia Diagnosis Rate (15–24-year-olds) Frequently Asked Questions (FAQ) 05/2013, 2012 http://www.chlamydiascreening.nhs.uk/ps/resources/Annual_Chlamydia_Diagnosis_Rate_FAQ_May_2013_FINAL_VERSION.pdf

[6] van Bergen J, Götz HM, Richardus JH, et al Prevalence of urogenital *Chlamydia trachomatis* increases significantly with level of urbanisation and suggests targeted screening approaches: results from the first national population based study in the Netherlands. Sex Transm Infect 2005;81:17–23. 10.1136/sti.2004.010173 15681716 PMC1763744

[7] Biello KB, Pettigrew MM, Niccolai LM Multiple chlamydia infection among young women: comparing the role of individual- and neighbourhood-level measures of socioeconomic status. Sex Transm Infect 2011;87:560–2. 10.1136/sextrans-2011-050185 21940727 PMC3438508

[8] Crichton J, Hickman M, Campbell R, et al Socioeconomic factors and other sources of variation in the prevalence of genital chlamydia infections: a systematic review and meta-analysis. BMC Public Health 2015;15:729 10.1186/s12889-015-2069-7 26224062 PMC4520210

[9] Public Health England. Tables 1–4: Chlamydia testing data for 15–24-year-olds in England, January to December 2012. Data are presented by patient residence, 2012.

[10] Department for Communities and Local Government. The English Indices of Deprivation 2010, 2010.

[11] Public Health England. Public Health England changes to chlamydia data collection and reporting, PHE, 2015.

