# Supplementary material for: Chlamydia diagnosis rate in England in 2012: an ecological study of local authorities
Source: Sex Transm Infect. 2016 Aug 31;93(3):226–8. doi: 10.1136/sextrans-2015-052441 (PMC5520241; doi:10.1136/sextrans-2015-052441)
Supplement: Supplementary table [file sextrans-2015-052441supp001.pdf]

Supplementary Material

Table S1: Description of coverage, positivity and chlamydia diagnosis rate by population size category and gender and unadjusted prevalence ratios for the association between achieving a chlamydia diagnosis rate ≥2,300 cases per 100,000 and population size for local authorities in England in 2012

**Population size**	**No. of LAs**	**LAs meeting recommendation**		**Coverage (%)**		**Positivity (%)**		**Chlamydia diagnosis rate per 100,000**		**Unadjusted PR (95% CI)**	**Test for trend p value**
		**N**	**%**	**Mean**	**SD**	**Mean**	**SD**	**Mean**	**SD**		
Women											
**1-5000**	68	8	11.76	26.16	7.58	6.63	1.35	1,708	526	0.16 (0.08-0.33)	
**5001-10000**	143	59	41.26	32.17	16.93	7.16	1.35	2,286	1,183	0.58 (0.43-0.78)	
**10001-15000**	45	29	64.44	36.83	12.19	7.64	1.20	2,811	1,029	0.90 (0.66-1.24)	
**15001-20000**	40	23	57.50	36.84	13.49	7.16	1.23	2,637	1,103	0.81 (0.56-1.15)	
**20001+**	28	20	71.43	39.25	14.17	7.54	1.29	2,943	1,191	Reference	<0.0001
Men											
**1-5000**	54	0	0	10.72	4.69	9.21	2.86	913	295	-	
**5001-10000**	150	11	7.33	14.00	10.04	9.16	2.92	1,154	645	0.76 (0.22-2.56)	
**10001-15000**	49	8	16.33	17.22	8.10	9.53	2.36	1,550	657	1.69 (0.48-5.88)	
**15001-20000**	40	6	15.00	17.14	8.93	9.07	2.49	1,504	846	1.55 (0.42-5.71)	
**20001+**	31	3	9.68	18.01	7.57	8.66	2.66	1,443	470	Reference	0.2619*

*LA – Local Authority; PR – prevalence ratio; 95% CI – 95% Confidence Interval; SD – Standard Deviation; *excluding population group 1-5000*
